# A new catalytic site functioning in antigen cleavage by H34 catalytic antibody light chain

**DOI:** 10.1038/s41598-022-23689-6

**Published:** 2022-11-10

**Authors:** Emi Hifumi, Tamami Nonaka, Hiroaki Taguchi, Taizo Uda

**Affiliations:** 1 Institute for Research Management, Oita University, 700 Dannoharu, Oita-Shi, Oita, 870-1192 Japan; 2 Research Center for GLOBAL/LOCAL Infectious Diseases, Oita University, 700 Dannoharu, Oita-Shi, Oita, 870-1192 Japan; 3 Faculty of Pharmaceutical Sciences, Suzuka University of Medical Science, 3500-3 Minamitamagaki-Cho, Suzuka, 510-0293 Japan; 4 Nanotechnology Laboratory, Institute of Systems, Information Technologies and Nanotechnologies (ISIT), 4-1 Kyudai-Shinmachi, Fukuoka, 879-5593 Japan

**Keywords:** Biochemistry, Biotechnology, Chemical biology

## Abstract

The cleavage reactions of catalytic antibodies are mediated by a serine protease mechanism involving a catalytic triad composed of His, Ser, and Asp residues, which reside in the variable region. Recently, we discovered a catalytic antibody, H34 wild type (H34wt), that is capable of enzymatically cleaving an immune-check point PD-1 peptide and recombinant PD-1; however, H34wt does not contain His residues in the variable region. To clarify the reason behind the catalytic features of H34wt and the amino acid residues involved in the catalytic reaction, we performed site-directed mutagenesis focusing on the amino acid residues involved in the cleavage reaction, followed by catalytic activity tests, immunological reactivity evaluation, and molecular modeling. The results revealed that the cleavage reaction by H34wt proceeds through the action of a new catalytic site composed of Arg, Thr, and Gln. This new scheme differs from that of the serine protease mechanism of catalytic antibodies.

## Introduction

Catalytic antibodies are superior to monoclonal antibodies because they have the unique ability to enzymatically digest antigens such as peptides^1–5^, antigenic proteins^6–14^, DNA^15–17^, and physiologically active molecules^18–21^. Antigen cleavage by a catalytic antibody has been shown to often occur via a serine protease-like mechanism, where a catalytic triad composed of His, Ser and Asp (or catalytic dyad (His and Ser) in a rare case) contributes to the catalytic cleavage reaction, as confirmed by site-directed mutagenesis studies^22,23^, X-ray crystallography^24^, and mass spectrometry^25^. The three residues, which are necessary in the catalytic site of serine proteases, mostly reside in the variable region of the antibody subunits including light and/or heavy chains.

Most catalytic antibodies with potential for practical application are produced from natural antibodies. However, catalytic antibody research for medicinal advancement faces multiple challenges. One is the structural diversity problem^26,27^, the second is lack of clarity regarding the importance of Pro95^28^, and the third is lack of understanding regarding optimum conditions for generating a highly active state. We have determined solutions to these important unsolved problems through our research on catalytic antibody light chain^28–32^. Nonetheless, in the future, it is necessary to efficiently produce a catalytic antibody with practical antigen-degrading activity against several antigens in a short time. Because many monoclonal antibodies have developed since the report by Koller-Milstein^33^, we have developed an innovative method for converting antibodies into catalytic antibodies^28^. The key step in this approach is deletion of the Pro95 residue in CDR-3 of the antibody kappa light chain. This technique has allowed the enzymization of several antibodies (or antibody kappa light chains). Interestingly, in certain antibodies, the Pro95 residue is absent in CDR-3 of the antibody light chain or is replaced with another amino acid residue. In this study, we used the H34 wild type (H34wt) light chain. H34wt enzymatically cleaves the immune checkpoint molecule PD-1, as well as recombinant PD-1 molecule^34^; however, H34wt does not contain the typical catalytic triad comprising the residues His, Ser, and Asp, as His is absent in the variable area of H34wt. The mechanism by which the H34wt clone cleaves PD-1 molecule is unclear. Additionally, although most kappa type antibody light chains contain the Pro95 residue in CDR-3, H34wt lacks Pro95 in this region; instead, the basic amino acid Arg95 is present in place of Pro95. Therefore, in this study, we aimed to identify the role of amino acid residues residing at the 95th position and its surroundings by developing several mutants using site-directed mutagenesis techniques focused on these amino acids, followed by catalytic activity tests, evaluation of reaction kinetics, and structural analysis by molecular modeling.

## Results

### Importance of Pro95 in antibody light chains

**Figure 1:**
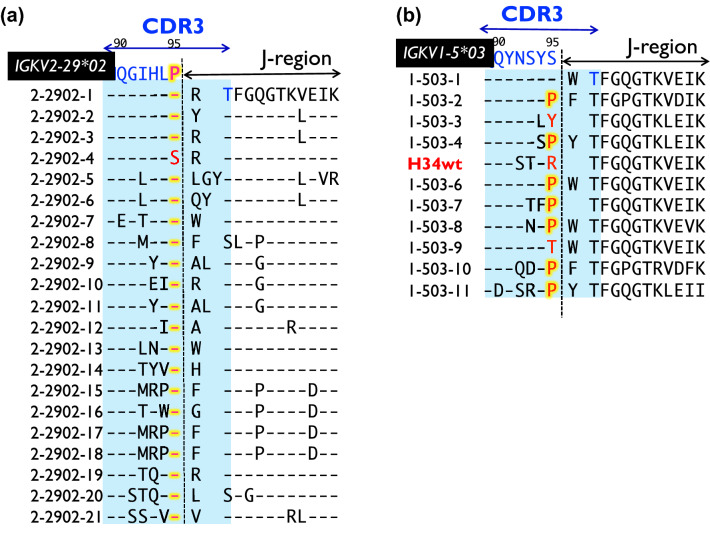
Comparison of amino acid sequences of CDR-3 among germline genes. (**a**) IGKV2-29*02: the amino acid sequences of germline gene IGKV2-29*02 and 21 human light chain clones. 20 out of 21 clones possess Pro95 residue at the 95th position, indicating that Pro95 is highly conserved. (**b**) IGKV1-5*03: *IGKV1*-5*03 does not contain Pro95; a Ser residue is placed at this position. Although the residue is replaced by somatic mutation, the Ser is replaced with Pro in 64% of the clones (seven out of eleven clones). Antibody light chains tend to contain Pro at the 95th position in CDR-3.

We have prepared some human and mouse antibody light chains of kappa-type and determined their amino acid sequences. Figure 1a and b present the amino acid sequences of CDR-3 in these human antibody light chains of kappa-type. Figure 1a presents the amino acid sequences of CDR-3 for 21 clones examined in this study for the human light chain encoded by the germline gene IGKV2-29*02 (IMGT classification. It is Subgroup II by Kabat’s classification). All clones except 2-2902-4 clone contain the Pro95 residue, suggesting that Pro95 is highly conserved (20 out of 21 clones; 95% of the clones). This tendency is also observed in other Vκ germline genes from IGKV1 to IGKV6 (see Fig. S1a–1c). Most clones retain proline residue locating at 95th (or the neighbor) in CDR-3. In contrast, the light chain encoded by the germline gene IGKV1-5*03 (Subgroup I by Kabat’s classification) does not contain Pro95, and the Ser residue is placed at the position of 95th of this protein (Fig. 1b). We randomly analyzed the amino acid sequences of 11 clones (1-503-5 clone was named as H34wt clone) of the human light chain encoded by the germline gene IGVK1-5*03, which were prepared in this study. Seven out of eleven clones contained somatic mutations, resulting in the placement of Pro at the 95th position. The rate of this mutation was 64%, which is very high, considering that 20 amino acids occur naturally in humans. This implies that Ser95 of the germline is predominantly mutated to Pro. These observations indicate that antibody light chains (kappa) tend to contain Pro95 in the CDR-3. The reason for this result is unknown at present. To investigate and clarify this phenomenon, we performed the following experiments, employing point mutagenesis focused on Pro95 and relevant residues.

1. H34wt
2. Amino acid sequence, preparation, SDS-PAGE analysis, and evaluation of catalytic features

**Figure 2:**
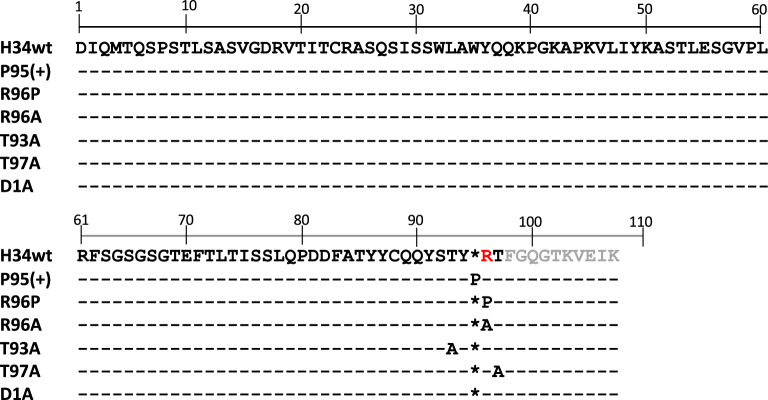
Amino acid sequences for H34wt and the mutants. The amino acid sequences of the variable region of H34wt and the mutants. There are no histidine residues in the variable region. Another feature of H34wt is the lack of Pro^95^, which is present in many antibodies. The amino acid residues considered to participate in the catalytic reaction are mutated to Pro or Ala. DNA sequences for H34wt and the mutants are deposited in DDBJ/GenBank/EMBL (see Data availability statement).

H34wt belongs to germline gene of IGKV1-5*03 (see Fig. S1a/red rectangular). Figure 2 presents the amino acid (aa) sequence of H34wt along with that of mutants prepared as per the procedures described in “Materials and methods”.

**Figure 3:**
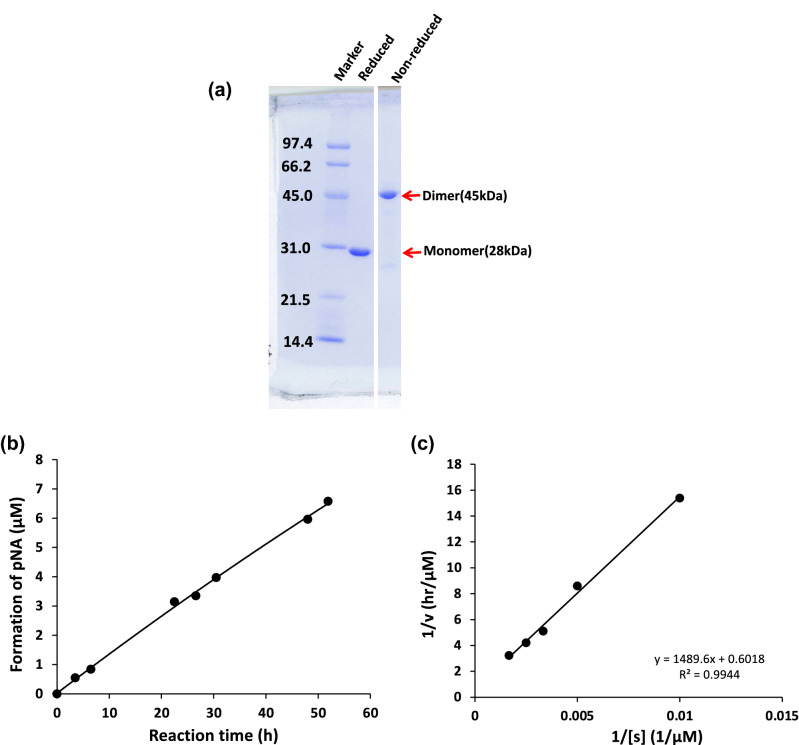
(**a**) Results of SDS-PAGE analysis for H34wt: SDS-PAGE analysis (12% gel) for H34wt after a size-exclusion chromatography is shown. A band of dimer at approximately 45 kDa is observed under the non-reduced condition. Under the reduced condition, only a single band of approximately 28 kDa is detected, which corresponds to the monomer. Bands other than the monomer of the light chains are essentially absent under either reduced or non-reduced condition. (**b**) Time course of the cleavage of the synthetic substrate Arg-pNA by H34wt. Reaction conditions: H34wt: 10 μM. Arg‐pNA: 200 μM. Reaction temperature: 37 °C. Reaction volume: 200 µL. Arg(R)‐pNA (trypsin‐like substrate) was used as the substrate in 50 mM/Tris-100 mM/Glycine-Tween-20 buffer (TGT) buffer. The reaction was performed in triplicate in a 96-well microplate. The figure shows that the cleavage reaction proceeds almost linearly depending on the reaction time. (**c**) Kinetics. Lineweaver–Burk plot for cleavage of Arg-pNA by H34wt. H34wt: 10uM. Arg-pNA: 100–800 µM. Reaction temperature: 37 °C. The plot presents a linear relationship, indicating that the cleavage reaction is enzymatic and in accordance with the Michaelis–Menten mechanism. Km = 2.48 × 10^−3^ (M) and kcat = 2.7 × 10^−3^ (/min).

The purity and molecular form of the purified H34wt were examined using SDS-PAGE analysis with Coomassie brilliant blue (CBB) staining (Fig. 3a). Under the non-reduced condition, the bands at approximately 45 kDa corresponded to the dimer of the H34wt light chain, suggesting that H34wt is present as the dimer form in the solution. Under the reduced condition, the bands observed at approximately 29 kDa corresponded to the monomer of the H34wt antibody light chain, whereas the other bands were barely observed, indicating the high purity of H34wt (> 95%).

To evaluate the peptidase activity of catalytic antibody light chain, Matsuura et al.^35^ and Durova et al.^8^ have used the substrate Arg-pNA and Pro-Phe-Arg.MCA, respectively. We employed the former synthetic substrate (Arg-pNA) to investigate the catalytic activity of H34wt. Figure 3b presents the reaction time course of H34wt-mediated digestion of Arg-pNA. Arg-pNA was gradually cleaved by H34wt in accordance with the reaction time elapsed. The kinetic study was performed by varying the concentration of Arg-pNA from 100 to 600 µM, while maintaining the concentration of H34wt constant at 10 µM. Figure 3c presents the Lineweaver–Burk plot, which demonstrates a linear relationship, indicating that the reaction obeys the Michaelis–Menten mechanism. The kcat and Km values were 2.7 × 10^−3^/min and 2.48 × 10^−3^ M, respectively.

1.2 Cleavage of FRET-PD1 peptide

**Figure 4:**
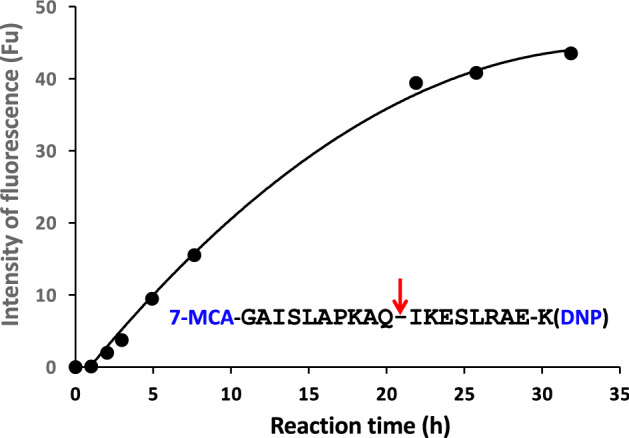
The time course for the cleavage of FRET-PD1 by H34wt: the cleavage reaction was performed with 5 µM H34wt using 25 µM of FRET-PD1 (PD-1; aa 123-140). The reaction was performed in duplicate. H34wt cleaves the FRET-PD1 peptide in a linear fashion up to 20 h of the reaction time, following which it reaches a plateau.

Figure 4 presents the reaction time course of H34wt-mediated digestion of the substrate FRET-PD1 (25 µM: aa 123rd–141st (7-MCA-GAISLAPKAQIKESLRAEK(DNP)-NH2)). Figure S2 presents the chemical structure of FRET-PD1 peptide. The digestion of FRET-PD1 gradually advanced for 20 h of the incubation, reaching a plateau after approximately 30 h of incubation. The peptide bond between Gln^132^ and Ile^133^ was cleaved by H34wt; this reaction was analyzed by means of HPLC and MS.

2. Mutants of H34wt

H34wt acts as a peptidase or/and protease, despite lacking a histidine residue in the variable region. Because H34wt contains arginine residues in the variable region, the suitably positioned arginine residue is possibly located in the catalytic site instead of histidine. Considering that, in H34wt, the Arg residue is located at the 95th position in the CDR-3, some mutations were introduced into the amino acid residues of CDR-3 of H34wt.

2.1 Six mutants prepared by site-directed mutagenesis

#### H34-Pro95(+)

We inserted Pro95 into H34wt; consequently, Arg95 moved to the 96th position in this mutant. Therefore, the amino acid number of the Arg residue is defined as 96 in order to maintain accuracy during comparison (Fig. 2). H34_Pro95(+) is represented as Pro95(+) below.

#### H34-R96P

Arg96 was mutated to Pro96 for investigating the effect of Pro96 and Arg96. H34_R96P is represented as R96P below.

#### H34-R96A

This mutant was prepared for investigating the effect of Arg96; Pro95 is absent in this mutant. H34_R96A is represented as R96A below.

#### H34-T97A & H34-T93A

In H34wt, several threonine residues are located close to the Arg95 residue. The threonine residue has a hydroxyl group, as does the serine residue. Thus, to investigate whether the hydroxyl group of the Thr residue involved in the catalytic activity, we prepared the mutants H34-T97A and H34-T93A. H34-T97A and H34-T93A are represented as T97A and T93A, respectively, below.

#### H34-D1A

In subgroup II of kappa light chains, Asp1 is frequently involved in catalytic reactions as a residue of the catalytic triad^3,4,7,22^. Therefore, the H34-D1A mutant was prepared for investigating the role of Asp1. H34_D1A is represented as D1A below.

2.2 SDS-PAGE for H34wt and its mutants

**Figure 5:**
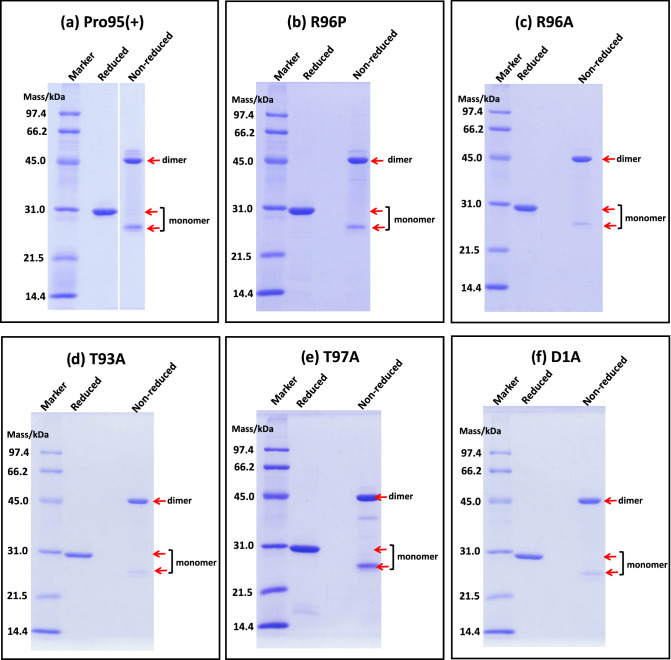
SDS-PAGE for mutants. SDS-PAGE (12% gel) with CBB staining was carried out following the procedure described in Fig. 3a. Each mutant was purified by cation chromatography. A band of dimer at approximately 45 kDa is observed under non-reduced condition. (**a**) Pro95(+); A faint band at ~ 50 kDa is observed over the dimer band at 45 kDa under non-reduced condition. Under the reduced condition, a band is observed at approximately 29 kDa; no other bands are detected. (**b**) R96P; In this sample, a faint band at ~ 50 kDa is observed over the dimer band at 45 kDa under non-reduced condition. Under the reduced condition, a band is observed at approximately 28 kDa; no other bands are detected. (**c**) R96A; Under both reduced and non-reduced conditions, no other bands except R96A mutant are observed. (**d**) T93A; Under both reduced and non-reduced conditions, no other bands except R96A mutant are observed. (**e**) T97A; A faint band at ~ 40 kDa below the dimer band (44 kDa) is detected under the non-reduced condition, whereas the monomer band is clearer compared with that of other mutants. A faint band caused by a small amount of impurities is observed at ~ 16 kDa. (**f**) D1A; under both reduced and non-reduced conditions, no other bands except R96A mutant are observed.

All mutants were purified using an Ni–NTA affinity column, followed by cation exchange chromatography. Figure 5a presents the results of SDS-PAGE analysis of Pro95(+) under reduced and nonreduced conditions. Under the nonreduced condition, we observed bands at approximately 45 and 27 kDa, which correspond to the dimer and monomer, respectively, as well as a faint band over 45 kDa. The monomer band at 27 kDa was clear, suggesting that the monomer was present in the solution. Under the reduced condition, only a 30-kDa band corresponding to the monomer was detected, and bands other than the monomer of the light chain were hardly observed, suggesting that the Pro95(+) light chain was highly purified.

Figure 5b–f present the results of SDS-PAGE for the purified mutants R96P, R96A, T93A, T97A, and D1A, respectively. The monomer band at approximately 26 kDa were detected in all SDS-PAGE gels under the non-reduced condition. Similar results were observed for all mutants examined. For T97A, the monomer band was clearer compared with that of other mutants and a low amount of impurities was detected as a faint band at ~ 16 kDa.

2.3 Cleavage tests for FRET-PD1 peptide

**Figure 6:**
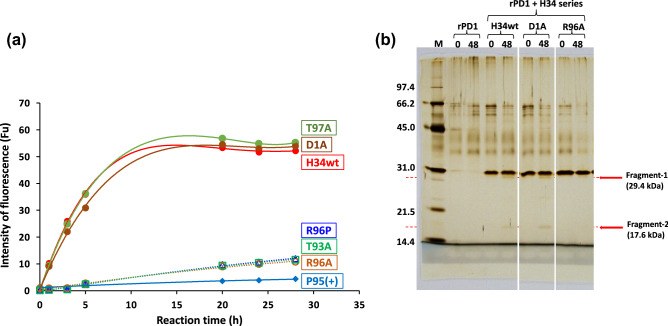
(**a**) Time course of the cleavage reaction for FRET-PD1 peptide by mutants. FRET-PD1: 25 µM. H34wt & mutants: 5 µM. In this experiment, H34wt was tested together with the mutants for ensuring accurate comparison among the samples. H34wt and the mutants of D1A and T97A show high catalytic activity. In contrast, three mutants of R95P, R95A, and T93A exhibit low catalytic activity. The catalytic activity of P95(+) is substantially suppressed. (**b**) Cleavage of recombinant PD-1 molecule. Recombinant PD-1 (rPD-1): 1 µM. H34wt, D1A, R96A and T97A: 0.5 µM. Reaction temperature: 37 °C. The SDS-PAGE (12% gel) experiment was performed under reduced condition, and the visualization was performed by silver staining. The cleavage reaction by H34wt and the several mutants were carried out over 48 h of incubation. The robust bands at approximately 29-kDa represent the monomer band of H34wt and/or the mutants. In H34wt light chain, a band at 17.5 kDa, which corresponds to the fragment of rPD1 (see ref.), is detected at 48 h of incubation; however, it is not detected at 0 h of incubation. Similar results are observed for D1A mutant, where the same band at 17.5 kDa is detected. In contrast, no band at 17.5 kDa is detected in the case of the mutant R96A, indicating that R96A does not cleave rPD-1. These results are consistent with the cleavage activity for FRET-PD1. Both H34wt and D1A, but not R96A, cleaves FRET-PD1 peptide with high activity.

We performed cleavage tests for all mutants using the FRET-PD1 peptide (whose structure is shown in Fig. S2) under the same reaction conditions presented in Fig. 4. The reaction time courses are shown in Fig. 6a; H34wt was tested again along with the mutants for ensuring accurate comparison among the samples used in the experiment. H34wt and the mutants T97A and D1A showed high catalytic activity. In contrast, the mutants R96P, R96A, and T93A exhibited low catalytic activity. The catalytic activity of the mutant P95(+) substantially reduced.

2.3.4 Cleavage of recombinant PD-1 molecule

A recombinant PD-1 molecule (rPD-1, 1 µM), the amino acid sequence of which is presented in Fig. S3, was mixed with H34wt or its mutants (0.5 µM) in a TGT buffer containing 0.02% sodium azide and incubated at 37 °C. The mutants of H34wt were created by replacing Asp1 and Arg96 with alanine. Samples collected after 0 and 48 h were analyzed by SDS-PAGE with silver staining. Figure 6b shows the results of the reaction of rPD-1 with H34wt and the mutants (D1A and R96A). H34wt and D1A, but not R96A, catalyze the cleavage of the FRET-PD1 substrate. In the figure, the clear bands positioned at 30.6, 30.1, and 30.8 kDa correspond to the light chains of H34wt, D1A, and R96A, respectively. For H34wt, other bands were not observed, except the band at 30.6 kDa. However, after 48 h of incubation, the bands at 29.4 kDa (Fragment 1) and 17.6 kDa (Fragment 2) could be detected. According to a previous report, the band at 17.6 kDa corresponds to a fragmented peptide from the N-terminus of rPD1 cleaved at Q^132^–I^133^^34^. In the case of D1A, a similar band was detected in the same position at 29.4 kDa (Fragment 1) and 17.6 kDa (Fragment 2) after 48 h of incubation. In contrast, in the case of R96A, the band at 17.6 kDa was absent, while the band at 29.5 kDa was faint. The catalytic activities of the above three clones for rPD-1 cleavage were consistent with that for FRET-PD1 peptide cleavage.

2.4 Kinetic constants

The kinetic constants of kcat and Km of each mutant were approximately estimated for FRET-PD1 cleavage reaction by using Michalis–Menten equation (Table 1). The kcat and Km values of H34wt were 6.6 × 10^−3^/min and 32.8 × 10^−6^ M, respectively. The mutants T97A and D1A showed kcat values of 11.5 × 10^−3^/min and 9.9 × 10^−3^/min, respectively. Thus, H34wt, T97A, and D1A were categorized as the high-activity group (Table 1). The mutants T93A, R96A, and R96P, which were classified as the low-activity group, exhibited kcat values of 0.2 × 10^−3^, 1.1 × 10^−3^, and 0.2 × 10^−3^/min, respectively. Those values were lower than that of the high-activity group by a factor of approximately one-tenth. The kcat of P95(+) was very low, implying that P95(+) almost lost its catalytic activity.

2.5 ELISA

**Table 1:** Kinetic constants of H34wt and the mutants for FRET-PD1 cleavage reaction.

Group	H34 series	kcat (/min) (× 10^–3^)	Km (M) (× 10^–6^)
High-activity	H34wt	6.6	32.8
	T97A	11.5	39.1
	D1A	9.9	40.5
Low-activity	T93A	0.2	31.1
	R96A	1.1	62.1
	R96P	0.2	33.6
Very low-activity	P95(+)	0.003	25.1

**Figure 7:**
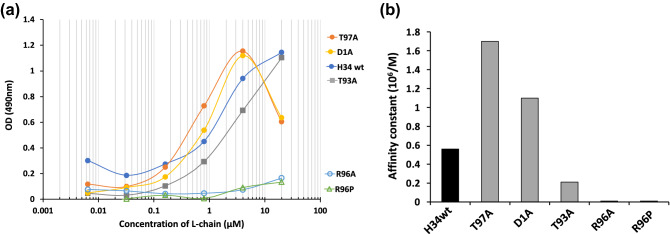
(**a**) ELISA: Coated antigen (rPD1): 2 µg/mL and 50 µL/well. Concentration of H34wt and the mutants: 0.0064–20 µM. Second antibody: POD-goat IGG to human Kappa chain (1/2000 dilution). H34wt shows the typical sigmoid curve of ELISA. Both T97A and D1A mutants shows the sigmoid curve with prozone phenomena. The mutants are divided into two groups. The first group including H34wt, T97A, D1A, and T93A shows binding affinity to rPD-1, while the second group including R96A and R96P hardly binds to rPD-1. This means that Arg96 is strongly involved in the recognition of rPD-1. (**b**) Comparison of affinity constants. The affinity constants, which were calculated from the results of (**a**), are presented for comprehensive comparison. It is clear that the replacement of The93 and Arg96 to Ala (or Pro) decreased the antigen recognition.

For investigating the immune affinity to recombinant PD-1 molecule (rPD-1), ELISA was performed for each light chain. The results are shown in Fig. 7a. H34wt showed a sigmoid curve, which is typically observed in the results of ELISA. T97A and D1A exhibited the typical prozone phenomenon beyond 4 µM of the catalytic antibodies in the ELISA. A strong binding affinity to rPD-1 was observed for T97A, D1A and H34wt. In contrast, the absorbance (OD) of R96A and R96P was very low. This implies that the amount of these mutants that bind to rPD-1 is low. Interestingly, the absorbance of T93A was comparable with that of the low-activity group (T97A, D1A, and H34wt); however, T93A (low-activity group) showed a considerably lower catalytic activity than the high-activity group. Figure 7b presents the affinity constants, which were calculated from the results of Fig. 7a, for several mutants.

2.6 Molecular modeling

The molecular modeling is not appropriate to understand with high accuracy. However, it is a useful tool as a guide to interpret the present results without X-ray diffraction analysis.

2.6.1 H34wt

**Figure 8:**
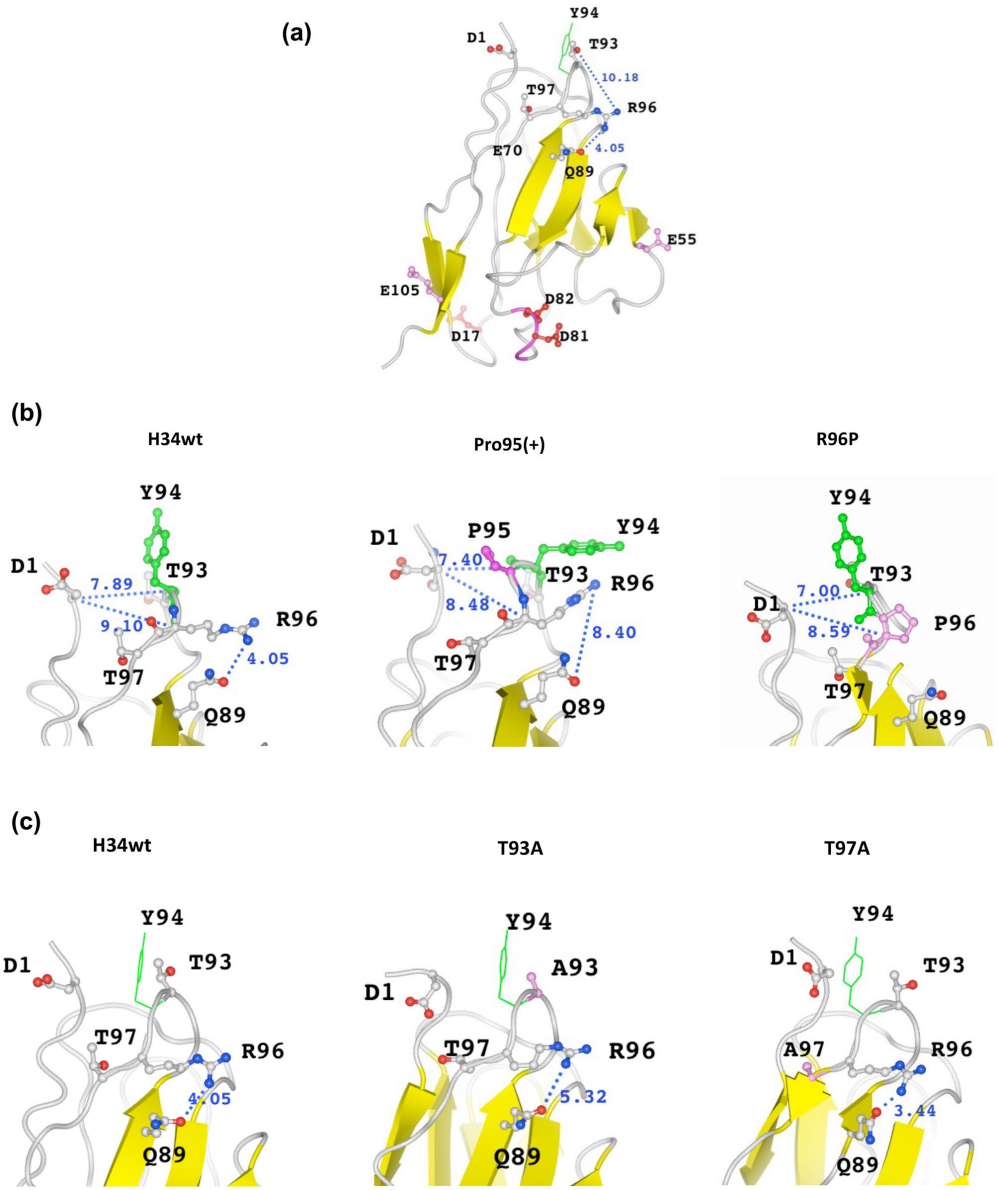
Molecular modeling. Numerical number is the distance between two atoms (Å). (**a**) H34wt: Asp17, Asp81, Asp82, Glu55, Glu70, and Glu105 residues capable of pulling a proton of Arg96 are present in the variable region; however, they are too far to interact with Arg96. The carbonyl of Gln89 residue can interact with the hydrogen of amino group of Arg96. The distance between them is 4.05 Å, which is preferable for interaction. (**b**) Comparison of H34wt, Pro95(+), and R96P: the conformation of Tyr94 residue of H34wt and Pro95(+) is considerably changed from the vertical position in H34wt to the horizontal position in Pro95(+). Additionally, the conformation of Arg96 is considerably altered. The conformational changes between H34wt and R96P are small, except the replacement of the residue. In H34wt, Pro(95+) and R96P, the distances of Cα between D1-T93 and D1-R96 are in the range within 1.0 Å, suggesting small changes in the clones. (**c**) Comparison of H34wt, T93A, and T97A: the conformations of Arg96 are slightly different among the three light chains; however, those of Tyr94 are not considerably changed. The distance between Q89(O)-R96(N) becomes longest (5.32 Å) in T93A among three clones. The β-sheet content appears to increase in the mutants T93A and T97A compared with that in H34wt.

Figure 8a–c show the structural models of the H34wt light chain and the mutants. Previous studies on the catalytic characteristics of antibody light chains^3,7,22,32^, indicate that the amino acid residues Asp1, Ser27a, and His93 (or His27d) are the residues most likely to form a catalytic triad-like structure. However, although, in the H34wt light chain, His residues are absent in the variable region, Arg96 exists in CDR-3 of the variable region. The His and Arg residues are basic amino acids, while Ser and Thr are polar hydrophilic amino acids.

On the basis of the catalytic activity of the mutants, as reported above, we assume that Arg96 and Thr93 act as the amino acid residues of the catalytic site in H34wt. Arg96 residue may participate as a basic amino acid instead of His, and Thr93 as a polar hydrophilic amino acid instead of Ser, according to the catalytic triad mechanism. Therefore, molecular modeling was performed with a focus on the structures of Arg96 and Thr93, as well as relevant amino acids (Fig. 8a). The Asp and Glu residues, which can pull a proton from Arg96, are present at the position of Asp17, Asp81, Asp82, Glu55, Glu70, and Glu105 in the variable region; however, they are too far to interact with Arg96. In contrast, the Gln89 residue is situated close to Arg96. While Gln is classified as a neutral amino acid, the carbonyl of the Gln89 residue can interact with the hydrogen of the amino group of Arg96. The distance between Arg96(N) and Gln89(O) is 4.05 Å, which may enable their interaction.

2.6.2 Pro95(+) and R96P

Modeling with Pro95(+) (insertion of Pro95) and R96P (replacement of Arg96 to Pro96) was performed in the same manner as stated above. The results are shown in Fig. 8b, along with the result for H34wt. The conformation of the Tyr94 residue of H34wt and Pro95(+) was considerably changed from the vertical (H34wt) to horizontal (Pro95(+)) position, suggesting that the insertion of the Pro95 residue had a huge effect on the structure. In contrast, the distances of Cα between Asp1-Thr93 and Asp1-Arg96 are in small changes within 1.0 Å difference. Furthermore, the distance between Arg96(N) and Gln89(O) was 8.40 Å, which is greater than that in H34wt. Replacement of Arg96 with Pro96 did not considerably change the conformation of Tyr94.

2.6.3 T93A and T97A

Figure 8c presents the mutants, T93A and T97A, along with H34wt. In these two mutants, the conformation of Tyr94 was not considerably changed from that in H34wt; however, the conformation of Arg96 in the T93A mutant changed slightly compared with that in H34wt and the T97A mutant. CDR-3 did not exhibit a big structural change, despite the presence of the point-mutated amino acid residues in this region. The mutation of Thr to Ala in the CDR-3 region did not significantly affect the CDR-3 structure; however, it may have affected the overall structure. Several β-sheets appeared in the FR-1, CDR-1, FR-2, and CDR-2 regions of T93A and T97A mutants. The β-sheet content of T93A and T97A was increased to 32.4% and 27.8%, respectively, relative to that of H34wt, which was 25.9%. The effect on the catalytic activity is unknown.

## Discussion

The absence (or deletion) of Pro95 is rarely observed in IGKV1 (subgroup I) of human kappa light chains. There are few reports on the importance of the Pro residue residing in the CDR-3 region of kappa-type light chains. Because the absence of Pro95 in the CDR-3 can considerably contribute to the expression and/or enhancement of catalytic properties of the antibody^28^, we inserted the Pro residue into the 95th position (P95(+)) and replaced Arg96 with Pro96 residue (R96P). The insertion of Pro95th (Pro95(+)) considerably reduced the catalytic activity in the cleavage of FRET-PD1 peptide (Fig. 6a). These results are consistent with that of a previous study^34^.

H34wt showed that the values of kcat and Km were 2.7 × 10^–3^/min and 2.48 × 10^–3^ M, respectively, for Arg-pNA cleavage. Matsuura et al. reported that kcat and Km was 7 × 10^–2^/min and 0.21 × 10^–4^ M, respectively, using the Bence Jones protein (MOR) taken from a multiple myeloma patient and Arg-pNA substrate^35^. Durova et al.^8^ found that kcat and Km was 1.55 × 10^–3^/min and 5.3 × 10^–4^ M, respectively, using the L12 light chain for the Pro-Phe-Arg-MCA. In our previous paper, the #7TR human catalytic antibody light chain showed kcat and Km values of 2.2 × 10^–2^/min and 5.35 × 10^–4^ M, respectively^32^. In the case of H34wt, the kcat was comparable value compared with those values reported. However, the value of Km was higher than the reported values by a factor of ten. On the other hand, for the cleavage of FRET-PD1 substrate, the kcat of P95(+) was 1000-fold lower than that of H34wt as presented in Table 1. Moreover, R96P showed a low cleavage activity, similar to the P95(+) mutant. On the basis of these results, we suggest that Pro95 (or Pro96) is important for the catalytic activity so as to reducing it.

Next, we consider the role of Arg96 in the catalytic reaction, because both Arg and His are basic amino acids and the His residue in CDR-3 is typically a component of the catalytic triad. The catalytic activity of R96P and R96A mutant was clearly reduced (Fig. 6a), and, according to ELISA, their binding affinity to rPD-1 was lowered. These results indicate that Arg96 is involved in not only the cleavage of the PD-1 peptide but also the recognition of the PD-1 antigen.

In the case of serine protease, an oxyanion hole is created using the hydroxyl group of the Ser residue^36^. In several catalytic antibodies reported till date, Ser27a is often used in CDR-1. Many Ser residues are present in the variable region of H34wt; however, all of them are far from Arg96. In H34wt, both Thr93 and Thr97 are located close to Arg96. As Ser and Thr residues belong to the same category of amino acids, polar hydrophilic amino acids, the two Thr residues were mutated to Ala (T93A and T97A). Consequently, the T93A mutant showed a huge reduction in catalytic activity however, the catalytic activity of T97A was not changed. These results indicate that Thr93 must be present in the catalytic site, while Thr97 is not necessary. Regarding the role of Thr, Sakae et al. reported that Thr17 contributed to the formation of the oxyanion hole in a cutinase-like enzyme (CLE)^37^. In our case, Thr93 of H34wt can be considered to play a similar role as that described by Sakae et al. In the molecular modeling studies, these two mutants did not exhibit considerable structural differences, even for residues such as Thr93, Thr97, Tyr94, Agr96, and Gln89 (Fig. 8c). Hence, the mutation of Thr93 to Ala93 is the crucial point in the reduction of catalytic activity, which implies that Arg96 and Thr93 constitute the catalytic site of H34wt. Furthermore, we consider the possibility of the catalytic triad; the Asp residue needs to be located close to Arg96 (and Thr93). In the variable region, four Asp residues are situated at the 1st, 17th, 81st, and 82nd positions. Asp1 is excluded as a candidate because it did not influence the catalytic activity of the mutant D1A (Fig. 6a). The Asp residues located at the 17th, 81st, and Asp82nd positions are too far from Arg96, according to the molecular modeling studies (Fig. 8a). Glu, which possesses a carboxyl group and belongs to the same category as Asp (acidic amino acid), is considered a preferable residue that acts similar to Asp. However, no Glu residues (E55, E70 and E105) are located within a suitable distance of Arg96 for interaction with the latter. Interestingly, Gln residues, which contain a carbonyl group, are located at the 89th and 90th positions. The distance between the carbonyl group of Gln89 and amino group of Arg96 is approximately 4.05 Å (see Fig. 8b/H34wt), which is suitable for interaction. Regarding the role of Gln, Sakae et al. performed a suggestive study in which Gln86 adjacent to Thr17 contributes to the stabilization of the oxyanion hole in the previous CLE study. These results indicate that Gln89 can participate in the generation of the catalytic triad in our study.

Additionally, these two residues (Arg96 and Thr93) can possibly generate a catalytic dyad and act similar to the His/Ser or His/Asp dyad reported by Kolesnikov et al.^25^, Liao et al.^38^ and Paetzel et al.^39^.

**Figure 9:**
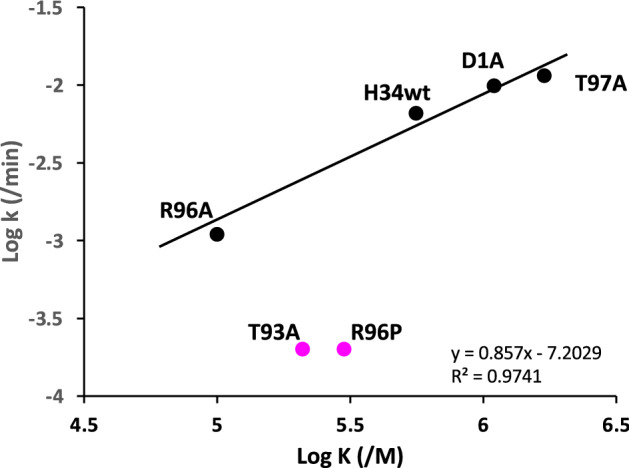
Linear Free Energy Relationship (LFER). LFER exists among the H34wt, T97A, D1A, and T93A clones, suggesting that features of these four clones are slightly different. In contrast, T93A and R96P are out of the linearity, indicating that the features of these two light chains must be hugely different from the above four clones.

For catalytic antibodies, we can determine two types of affinity constants for the antigen. One is the reverse of the Michaelis constant (1/Km), obtained by kinetic analysis. Another is the equilibrium constant (K), obtained through an immunochemical experiment such as ELISA. The value obtained using ELISA represents the binding affinity for the antigen recognition site of the antibody. In contrast, 1/Km represents the binding affinity for the catalytic site. When these two values are in good agreement, the binding site and the recognition site is identical. However, when these values are considerably different, the two sites are located in different parts^40^. The values obtained in this study are summarized in Table 2; the affinity constants obtained by ELISA (K) and the kinetic experiment (1/Km) are different by one or two orders of magnitude. This implies that the antigen recognition site and active site are not identical but located in different parts. In addition, the plots of log kcat vs log K (ELISA) for H34wt, T97A, D1A, and T93A showed a good linearity (Fig. 9). This indicates that a linear-free-energy relationship (LFER;^41–43^) exists among these four light chains, suggesting that the features of the wild type and the three mutants are not considerably different. On the contrary, T93A and R96P are out of the linearity, indicating that the chemical features of these two light chains are drastically changed compared with the above four light chains. Thr93 is an essential component of the catalytic site, and it interacts with Arg96. Replacement of Arg96 with Pro96 causes a huge structural modification. This is consistent with the observation that Arg96 and Thr93 are inevitable for the catalytic activity of H34wt.

**Table 2:** Affinity constants obtained by two methods.

H34 series	1/Km (kinetics) (× 10^5^/M)	K (ELISA) (× 10^5^/M)
H34wt	0.33	5.6
T97A	0.26	17
D1A	0.25	11
T93A	0.32	~ 2.1
R96A	0.16	~ 1
R96P	0.30	~ 3

**Figure 10:**
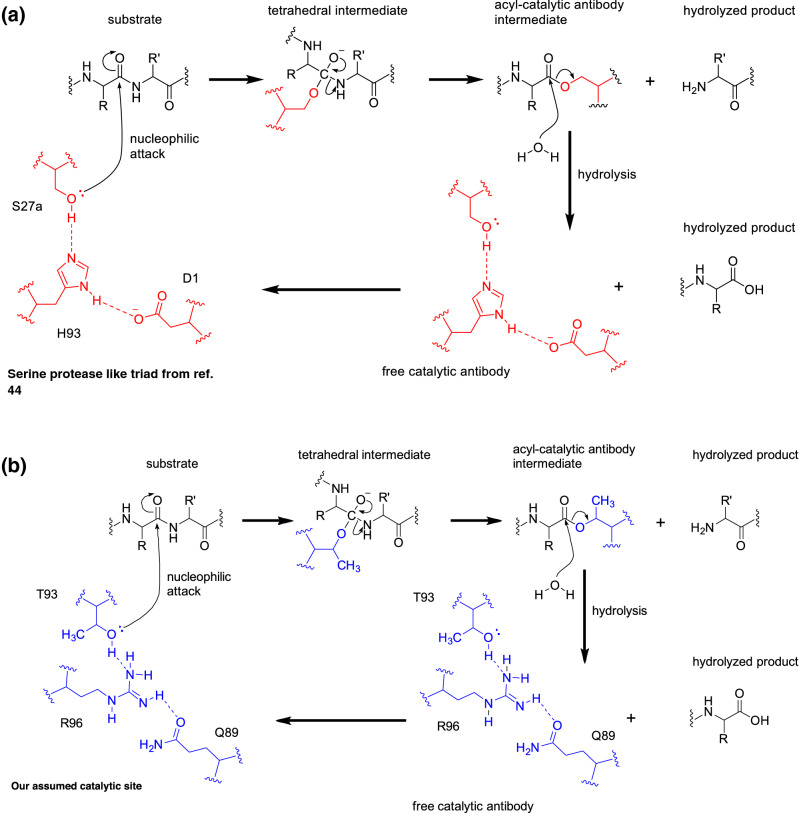
Reaction scheme by serine protease-like triad and catalytic site of H34wt. (**a**) In the case of serine protease-like triad, the hydroxyl group of the catalytic Ser of the catalytic antibody directly initially attacks the cleavable bond. (**b**) In the case of H34wt, similarly, the hydroxyl group of the catalytic Thr of H34wt directly attacks the scissile bond. Then, finally, the deacylation of the acyl catalytic antibody intermediate with water forms a second product and regenerates the free catalytic antibody.

Taking these observations into account, the reaction scheme for the hydrolysis of catalytic antibody with serine protease-like triad and mutant H34wt assumed is depicted in Fig. 10a and b. The serine protease-like triad is consisted of Ser-His-Asp. On the other hand, the mutant H34wt is consisted of Thr-Arg-Gln. Initially, the hydroxyl group of the catalytic Ser of the catalytic antibody directly attacks the cleavable bond (Fig. 10a)^44^. Similarly, the hydroxyl group of the catalytic Thr of H34wt directly attacks the scissile bond (Fig. 10b). Deacylation of the acyl catalytic antibody intermediate with water forms a second product and regenerates the free catalytic antibody.

**Figure 11:**
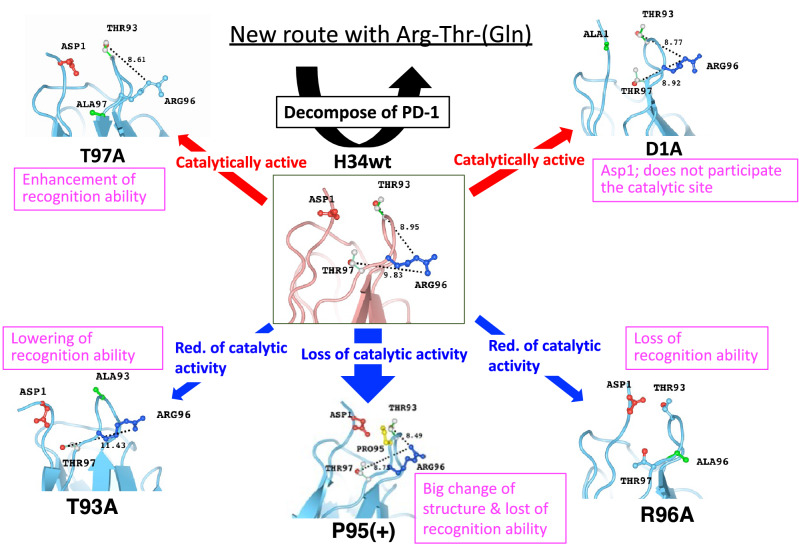
Interrelations of features of H34wt and the mutants. Mutagenesis in this study clarified the amino acid residues constructing the catalytic site, which is composed of Arg96, Thr93, and Gln89, revealing a new catalytic site for the cleavage of PD-1, which is different from the serine protease triad. The figure shows the interrelation among H34wt and the mutants; in addition to structural changes, several crucial changes are detected in terms of chemical and immunological characters. The number indicates the distance between the Arg and Thr residues. The figure shows the interrelation among H34wt and the mutants; in addition to structural changes, several crucial changes are detected in terms of chemical and immunological characters. The number indicates the distance between the Arg and Thr residues.

Interrelations of features among H34wt and the mutants, as investigated in this study, are summarized in Fig. 11. The clones (T97A and D1A), indicated with red arrows, maintain the catalytic activity. The molecular recognition for rPD-1 by the former clone was enhanced. In the latter clone, Asp1 was not a component of the catalytic site. The clones (T93A, Pro(95+) and R96A), indicated with blue arrows, reduced or almost lost their catalytic activity. In T93A, the catalytic activity was substantially reduced, and its ability to recognize PD-1 was lowered. Pro95(+) showed a drastic transformation of its features; the molecular structure was considerably different from that of H34wt, resulting in the loss of catalytic activity, as well as the ability to recognize PD-1. In the R96A clone, the ability for molecular recognition was lowered, along with reduction in catalytic activity.

These observations and discussion indicate a new catalytic site for H34 catalytic-antibody-mediated antigen cleavage that involves a Thr93–Arg96 residues (like-dyad) or a Gln89–Thr93–Arg96 residues (like-triad), different from Asp-Ser-His triad. As many antibody light chains belonging to IGKV1 contain the residues Gln89, Ser93, and Pro95, it is considered that the light chain can be easily converted into a catalytic light chain by the replacement of Pro95 to Arg95. The newly generated catalytic site or route can act to cleave the antigens. This finding can immensely contribute to the development of catalytic antibodies.

## Materials and methods

### Reagents

Chemical reagents such as Tris, glycine, CuCl_2_·2H_2_O, KCl, Na_2_HPO_4_·12H_2_O, NaCl, KH_2_PO_4_, EDTA·2Na and IPTG were purchased from Wako Pure Chemical Industries Ltd., Osaka, Japan (Guaranteed Reagent). The synthetic substrate peptidyl-pNA, Arg-pNA, was purchased from Peptides Institute Inc., Osaka, Japan. Tryptone and yeast extract were purchased from Becton–Dickinson and Company, NJ, USA. A commercially available recombinant PD-1 molecule was used (ENZO Life sciences Inc., Product Number; ENZ-PRT190; PD-1 (aa 25-167) containing a 5′-His-tag, V5 epitope tag spacer, and FLAG-tag.

### Synthesis of FRET substrates

The FRET-PD1 peptide (7-MCA-GAISLAPKAQIKESLRAE-K(DNP)-NH_2_; PD1 aa123-140) were synthesized on solid support using Fmoc/tBu strategy on the Rink amide resin as previously reported^45^. Briefly, removal of Fmoc group was carried out with 20% piperidine in dimethylformamide, whereas the chain elongation was achieved with standard HBTU/HOBt chemistry using 3 equivalents of protected amino acids or 7-MCA. After completing the synthesis, the protected peptide resin was treated with TFA/phenol/H_2_O/thioanisole/1,2-ethanedithiol (82.5:5:5:2.5, v/v/v/v) mixture. The crude material obtained was purified by HPLC. The structures of FRET peptides were confirmed by MS.

### Amplification of DNA fragments encoding light chains

Preparation of the H34wt gene was obtained in accordance with that described in references^32,34^.

Briefly stated, peripheral blood lymphocytes obtained from healthy volunteers were harvested using a Ficoll-Paque (GE Healthcare UK Ltd., Buckinghamshire, England) gradient, and stored appropriately. Total RNA was prepared from 3.0 × 10^7^ cells using an RNA isolation kit (Stratagene, La Jolla, CA, USA) for synthesizing cDNA. Oligo (dT) was used for reverse transcription PCR using the total RNA as a template (ThermoScript RT-PCR System; Invitrogen, Carlsbad, CA, USA). To prepare human antibody light chain belonging to IGKV1 (by IGMT classification), we used four mixed primers as forward primers

- 5′-CTACCATGGACATCCAGATGACCCAG-3′
- 5′-CTACCATGGACATCCAGTTGACCCAG-3′
- 5′-CTACCATGGCCATCCGGATGA CC-3′
- 5′-CTACCATGGTCATCTGGATGACCCAG-3′
  (a:b:c:d) = (46:2.5:1:0.5; mole ratio)

and a reverse primer 5′-GTACTCGAGACACTCTCCCCTGTTGAAG-3′. The PCR reaction occurred under the following incubation conditions: 30 s at 98 °C, 24 cycles of 10 s at 98 °C for denaturation, 30 s at 64.1 °C for annealing, and 30 s at 72 °C for extension. Finally, the extension was carried out for 5 min at 72 °C. In the PCR, Phusion (High-Fidelity DNA Polymerase, Finnzyme, Finland), was used. The amplified DNA fragment was inserted to pCR4Blunt-TOPO vector (Invitrogen, Zero Blunt TOPO PCR Cloning kit) and transformed to DH5α (TOYOBO). One hundred and fourteen colonies of DH5α were picked up and subjected to DNA sequencing. Eleven clones out of 114 clones were belonging to IGVK1-5*03 germline gene as presented in Fig. 1b, in which H34wt was included. The plasmid pCR4Blunt-TOPO was digested by *Nco* I and *Xho* I (New England Bio Lab) and inserted to pET20b (+) vector (Novagen, Madison, WI, USA), which was repurified and transformed into BL21 (DE3) pLysS for expression of H34wt light chains. Regarding preparation of human antibody light chain belonging to IGKV2 (in Fig. 1a), the details are explained in references^9,28^.

### Site-directed mutagenesis

In order to insert a point mutation to H34wt light chain, the following mutants were produced by site-directed mutagenesis.

#### Construct of H34_Pro95(+)

Deletion of Pro95 from the wild type of H34wt was performed by the method of inverse PCR using as the reverse primer 5′-CGGGTAGGTACTATACTGTTGGCAGTAATA-3′ and the forward primer 5′-CGGACGTTCGGCCAA-3′. In the experiment, KOD-Plus-Mutagenesis Kit (TOYOBO, Code SMK-101, Osaka) was used and the construct was firstly transformed to DH5α and finally to BL21(DE3)pLysS for the expression.

Following mutants were produced by the same method as mentioned above.

#### Construct of H34_R96P

Replacement of Arg96 to Pro was carried out by the same method stated above. The primers used in the inverse PCR were the reverse primer 5′-ATAAGTACTATACTGTTGGCAGTAATA-3′ and the forward primer 5′-ACGTTCGGCCAAGGG-3′.

#### Construct of H34_R96A

Replacement of Arg96 to Ala was carried out by the same method stated above. The primers used in the inverse PCR were the reverse primer 5′-CGCATAAGTACTATACTGTTGGCAGTA-3′ and the forward primer 5′-ACTTTCGGCCAAGGGAC-3′.

#### Construct of H34_T93A

Replacement of Thr93 to Ala was carried out by the same method stated above. The primers used in the inverse PCR were the reverse primer 5′-CGCACTATACTGTTGGCAGTAATAAGT-3′ and the forward primer 5′-TATCGGACGTTCGGCC-3′.

#### Construct of H34_T97A

Replacement of Thr97 to Ala was carried out by the same method stated above. The primers used in the inverse PCR were the reverse primer 5′-CGCCCGATAAGTACTATACTGTTGG-3′ and the forward primer 5′-TTCGGCCAAGGGACC-3′.

#### Construct of H34_D1A

Replacement of Asp1 to Ala was carried out by the same method stated above. The primers used in the inverse PCR were the reverse primer 5′-CATGGCCATCGCCGG-3′ and the forward primer 5′-GCGATCCAGATGACCCAGTCTC-3′.

### Sequencing

The H34wt and the mutants were sequenced with a ABI 3730xl Analyzer (Applied Biosystems, CA, USA) by using ABI BigDye™ Terminator v3.1 Cycle Sequencing Kits. GENETIX Ver. 8 (GENETIX, Tokyo, Japan) software was used for sequence analysis and deduction of amino acid sequences.

### Molecular modeling

Computational analysis of the antibody structures was performed using the deduced antibody light chain amino acid sequences by Discovery Studio (Accelrys Software, San Diego, CA, USA). For the homology modeling, the template structures were made by a BLAST search, following the minimization of the total energy of the molecule by using the CHARMM algorithm. The resulting Protein Data Bank (PDB) data were used for modification of the CDR (complementarity-determining region) structures defined by the Kabat numbering system.

### Culture, recovery and purification

The transformant was grown at 37 °C in 1 L of Luria–Bertani medium containing 100 μg/mL ampicillin to an A600 nm of 0.6 and then incubated with 0.01 mM IPTG for 20 h at 18 °C. Cells were harvested by centrifugation (3500 g, 4 °C, 10 min) and then resuspended in a 100 mL solution of 250 mM NaCl, 25 mM Tris-HCl, pH 8.0). The cells were lysed by ultra-sonication three times for 2 min each in an ice bath, followed by centrifugation (21,475 g, 4 °C, 20 min). The expressed human light chain was recovered as the supernatant.

The supernatant was first subjected to Ni-NTA column chromatography (Takara, Otsu, Japan) equilibrated with 25 mM Tris-HCl, pH 8.0, containing 250 mM NaCl. Elution was performed by increasing the concentration of imidazole from 0 and/or 30 to 300 mM. After the Ni-NTA column chromatography was completed, an aliquot of a solution of 50 μM CuCl_2_ (1.25 eq for the light chain) was added into the eluent (this is important to make a uniform (dimer) structure), based on the calculation that the absorbance of A600 nm of 1.0 in UV/VIS was regarded as ~ 1 mg/mL (40  μM light chain). Then, the solution including the light chain and copper ion was dialyzed against a 50 mM Tris-HCl buffer, pH 8.0, for about 20 h. After removing some aggregates by centrifugation (17,800 g, 4 °C, 20 min), the solution was concentrated to 2 mg/mL and subjected to a cation-exchange chromatography using a column of SP-5PW (TOSOH, Japan) with a gradient of NaCl (from 100 to 500 mM) in a 50 mM Na acetate buffer, pH 5.5, on the purification apparatus (AKTA system, GE-Healthecare-Japan, Tokyo) or subjected to a size-exclusion chromatography (column; HiLoad^TM^16/60 Superdex™ 200 pg (GE healthcare)) on the AKTA system, with a PBS (pH 7.4) buffer including 137 mM NaCl used as the eluent solvent. Then, the eluent was recovered and submitted to dialysis against 20 mM Tris-HCl/150 mM NaCl buffer (pH 8.5) for about 17 h, followed by concentrating the solution using Amicon ultra10000 (Millipore, USA). EDTA was put into the solution to be 50 mM and allowed to react for 1 h under the condition of 4 °C, followed by dialysis against 2 L of PBS, twice. After confirming the complete removal of Cu(II) by UV/VIS spectroscopy, it was filtered using a 0.2 µm membrane filter (Merck-Millipore) and stored at 4 °C or frozen. Protein concentrations were determined by the Bradford method using a Lowry method using the DC protein assay kit (Bio-Rad).

### SDS-PAGE analysis

Purified catalytic antibodies were submitted to SDS-PAGE analysis for investigating the purity. Resolving gel (12% gel) was prepared by mixing 45% acrylamide solution (2.64 mL), 1.5 M Tris–HCl (2.5 mL; pH = 8.8), pure water (4.2 mL), 10%SDS (0.1 mL), 10%APS (Ammonium persulfate/0.1 mL) and TEMED (*N*,*N*,*N*′,*N*′-tetramethylethlenediamine/10 μM). Stacking gel (5%-gel) was prepared by mixing 45% acrylamide solution (0.55 mL), 0.5 M Tris–HCl (1.25 mL; pH = 8.8), pure water (3.1 mL), 10%SDS (0.05 mL), 10%APS (0.05 mL) and TEMED (5 μM).

The solution of resolving gel was firstly put into the glass-made gel cassette, followed by the stacking gel solution. A 16-well comb was set at the upper part of the cassette. And it was allowed for 20–30 min till the completion of the polymerization of the solution and the sample (protein) was placed into each well. Then the cassette was equipped with an SDS-PAGE analysis apparatus (ATTO Model AE-8750, Tokyo), which was working for about 90 min under the initial condition of 250 V and 20 mA.

After the completion of the process, the gel was soaked with CBB (Coomassie brilliant blue) solution for visualization. In the case of the low content of protein, a silver-staining kit (WAKO Chemicals, Osaka) was used.

The part of stacking gel is no-need for the analysis. Frequently, the part is cut off with a knife from the original gel after the electrophoresis experiment. In this case, the edge of the upper part of gel is not seen, but the upper-top edge and the bottom-top edge of the resolving gel could be seen.

### Cleavage assays

To avoid contamination in cleavage assays, most glassware, plastic-ware, and buffer solutions used in this experiment were sterilized by heating (180 °C, 2 h), autoclaving (121 °C, 20 min), or filtration through a 0.20- μm sterilized filter, as much as possible. Most of the experiments were performed in a biologic safety cabinet to avoid airborne contamination.

#### For R-pNA substrate

Cleavage of the amide bond linking *p*‐nitroanilide to the C‐terminal aa in R‐pNA substrates (Peptides Institute Inc, Osaka, Japan) was measured at 37 °C in a 100 mM Glycine/50 mM Tris-HCl buffer containing 0.025% Tween20 (TGT buffer; pH 7.7) in 96‐well plates (96‐well plate/353075, Becton‐Dickinson, NJ, USA). The purified light chain (20 μL) was mixed with 180 μL of a synthetic substrate, R‐pNA. The final concentrations of the light chain and the substrate were 10 μM and 200 μM, respectively. Para‐nitroaniline formed from the substrate catalyzed by the light chains was detected by the measurement of absorbance at 405 nm, while 620 nm was employed as the reference using a microplate reader (Scanlt 3.1 for Multiskan FC, ThermoFisher Scientific, MA, USA). The peptidase activity of catalytic antibodies was estimated from the concentration of formation of *p*‐nitroaniline.

#### For FRET-PD1 peptide

FRET-PD1 (25 µM) was incubated with H34wt and the mutants (5 µM) in TGT buffer containing 0.02% NaN_3_ at 37 °C. Fluorescence was measured periodically on the Fluoroskan Ascent (λ_ex_ = 320 nm and λ_em_ = 405 nm; Thermo Fisher Scientific Oy, Vantaa, Finland). All measurements were done in duplicate.

#### For a recombinant PD1

Recombinant human type PD-1 molecule (1 µM: Enzo Life Sciences, ENZO-PRT-190, Farmingdale, NY)) was mixed with the purified H34wt and the mutants (0.5 µM) in TGT buffer including 0.02% NaN_3_ and incubated at 37 °C. Then they are analyzed SDS-PAGE with silver staining under reduced condition.

### Kinetics

#### For Arg-pNA

The concentration of H34wt light chain was fixed at 10 µM and that of the Arg(R)-pNA substrate was varied from 100 to 800 µM at 37 °C in the TGT buffer (pH 7.7). The concentration changes of R-pNA substrate within 10% conversion after mixing the H34wt light chain and the substrate was regarded as the initial rate of the reaction.

#### For FRET-PD-1

By integrating Michaelis–Menten equation, the plots for (2.3/t)*log([S]_0_/([S]_0_−[P])) vs [P]/t were taken. t: reaction time, [S]_0_: initial concentration of FRET-PD1, [P]; concentration of products.

From the slope, 1/Km was calculated and from the intercept, V/Km was. These values were obtained by using the reaction time courses in Fig. 6a.

### Enzyme-linked immunosorbent assay (ELISA)

50 μL of rPD-1 molecule dissolved in PBS solution (2 μg/mL) was fixed on a 96-well plate (Thermo Scientific, Denmark) at 4 °C overnight. Blocking was performed using 100 μL of 10% PBS solution of HCF (hybridoma cloning factor) for 30 min at room temperature. After the plate was washed with PBS-T, H34wt or the mutants was immunoreacted, followed by a reaction with anti- goat IGG to human Kappa chain conjugated with horse radish peroxidase (MP Biochemicals, OH, USA. Lot#;07697). After the substrate reaction was performed with 0.01% H_2_O_2_ including *p*-nitrophenyl phosphate (Sigma) dissolved in a 0.1 M citric acid/0.2 M phosphate buffer (pH 5.0), the reaction was stopped with 2 N H_2_SO_4_. Then, the absorption band at 490 nm (and 620 nm as the reference) was measured by use of a 96-well plate-reader (Becton‐Dickinson, NJ, USA).

### Statistical analysis

Statistical analysis such as correlation analysis was performed using a software of Microsoft Excel for Mac version 16.65.

## Supplementary Information

Supplementary Figures.

## Data Availability

All data needed to evaluate the conclusions in the paper are present in the paper and/or the Supplementary Materials. Additional data related to this paper may be requested from the corresponding author on reasonable request. DNA datasets of H34wt and the mutants generated and/or analyzed during the current study are available in the [DDBJ/GenBank/EMBL] repository (http://getentry.ddbj.nig.ac.jp/). The [accession number] of H34wt is [LC716350], P95(+) [LC716351], R96P [LC716354], R96A [LC716353], T93A [LC716355], T97A [LC716356] and D1A [LC716352]).

## Abbreviations

aa: Amino acid
AMC: 7-Amino-4-methylcoumarin
au: Arbitary unit
CBB: Coomassie brilliant blue
DNP: 2,4-Dinitrophenyl
Eq: Equivalent
FRET: Förster resonance energy transfer
IPTG: Isopropyl-β-d-thiogalactopyranoside
mAb: Monoclonal antibody
7-MCA: (7-Methoxycoumarin-4-yl)acetyl
MS: Mass spectroscopy
PBS: Phosphate buffered saline
PD1: Programmed cell death-1
TGT: 50 mM/Tris-100 mM/Glycine-Tween-20 buffer
TFA: Trifluoroacetic acid
