# Supplementary material for: A new catalytic site functioning in antigen cleavage by H34 catalytic antibody light chain
Source: Sci Rep. 2022 Nov 10;12:19185. doi: 10.1038/s41598-022-23689-6 (PMC9649737; doi:10.1038/s41598-022-23689-6)
Supplement: Supplementary file 1 — Supplementary Figures. [file 41598_2022_23689_MOESM1_ESM.pdf]

Supplemental Figures

Fig. S1 Classification of germline gene for human antibody kappa light chain by IMGT and the aa sequence of CDR-3

a) Germline genes IGKV1 (modified from IMGT Data base)
b) Germline genes IGKV2 and IGKV3 (modified from IMGT Data base)
c) Germline genes IGKV4, IGKV5 and IGKV6 (modified from IMGT Data base)
(Background of light blue indicates the head of subgroup)

Fig. S2 Chemical structure of FRET-PD1

FRET was adducted for the peptide from 123 to 140 of PD-1 (this region is the epitope of anti PD-1 mAb, Nivolumab). For the peptide, MCA (4-methyl-coumaryl-7-amide; Fluorescent reagent) was adducted at N-terminal and DNP (2,4-dinitrophenyl; Quenching reagent). Lysine was inserted in order to bind with DNP.

Fig. S3 AA sequences of human PD-1 and recombinant PD-1

Fig. S4a and S4b Explanations of SDS-PAGE gels

a) This shows the identical image as that of Fig. 3a
b) This figure explains the position of stacking gel, resolving gel, edge of upper and bottom-top lines. Frequently, the part of stacking gel is cut-off from the native gels because of no-need for analysis.

IGKV1		
IMGT gene name	IMGT allele name	aa of CDR-3
IGKV1-5	1-5*01	QQYNSYS
IGKV1-5	1-5*02	QQYNSYS
IGKV1-5	1-5*03	QQYNSYS
IGKV1-6	1-6*01	LQDYNYP
IGKV1-6	1-6*02	LQDYNYP
IGKV1-8	1-8*01	QQYYSYP
IGKV1D-8	1D-8*01	QQYYSFP
IGKV1D-8	1D-8*02	QQYYSFP
IGKV1D-8	1D-8*03	QQYYSFP
IGKV1-9	1-9*01	QQLNSYP
IGKV1-12	1-12*01	QQANSFP
IGKV1-12	1-12*02	QQANSFP
IGKV1D-12	1D-12*01	QQANSFP
IGKV1D-12	1D-12*02	QQANSFP
IGKV1-13	1-13*02	QQFNSYP
IGKV1D-13	1D-13*01	QQFNNYP
IGKV1D-13	1D-13*02	QQFNSYP
IGKV1-16	1-16*01	QQYNSYP
IGKV1-16	1-16*02	QQYNSYP
IGKV1D-16	1D-16*01	QQYNSYP
IGKV1D-16	1D-16*02	QQYNSYP

IGKV1		
IMGT gene name	IMGT allele name	aa of CDR-3
IGKV1-17	1-17*01	LQHNSYP
IGKV1-17	1-17*02	LQHNSYP
IGKV1-17	1-17*03	LQHNSYP
IGKV1D-17	1D-17*01	LQHNSYP
IGKV1-27	1-27*01	QKYNSAP
IGKV1-33	1-33*01	QQYDNLP
IGKV1D-33	1D-33*01	QQYDNLP
IGKV1-37	1-37*01	QQYDNLP
IGKV1D-37	1D-37*01	QRTYNAP
IGKV1-39	1-39*01	QQSYSTP
IGKV1D-39	1D-39*01	QQSYSTP
IGKV1D-42	1D-42*01	KQDFSYP
IGKV1D-42	1D-42*02	KQDFSYP
IGKV1D-43	1D-43*01	QQYYSTP
IGKV1-NL1	1-NL1*01	QQYYSTP

Fig. S1a

IGKV2		
IMGT gene name	IMGT allele name	aa of CDR-3
IGKV2-24	2-24*01	MQATQFP
IGKV2D-24	2D24*01	TQATQFP
IGKV2D-26	2D-26*01	
IGKV2D-26	2D-26*02	MQDAQDP
IGKV2D-26	2D-26*03	MQDAQDP
IGKV2-28	2-28*01	MQALQTP
IGKV2D-28	2D-28*01	MQALQTP
IGKV2-29	2-29*02	MQGIHLP
IGKV2-29	2-29*03	MQGIHLP
IGKV2D-29	2D-29*01	MQSIQLP
IGKV2D-29	2D-29*02	MQSIQLP
IGKV2-30	2-30*01	MQGTHWP
IGKV2-30	2-30*02	MQGTHWP
IGKV2D-30	2D-30*01	MQGTHWP
IGKV2-40	2-40*01	MQRIEFP
IGKV2-40	2-40*02	MQRIEFP
IGKV2D-40	2D-40*01	MQRIEFP

IGKV3		
IMGT gene name	IMGT allele name	aa of CDR-3
IGKV3-7	3-7*01	QQDHNLP
IGKV3-7	3-7*02	QQDYNLP
IGKV3-7	3-7*03	QQDHNLP
IGKV3-7	3-7*04	QQDYNLP
IGKV3-11	3-11*01	QQRSNWP
IGKV3-11	3-11*02	QQRSNWP
IGKV3-15	3-15*01	QQYNNWP
IGKV3-20	3-20*01	QQYGSSP
IGKV3D-7	3D-7*01	QQDYNLP
IGKV3D-11	3D-11*01	QQRSNWH
IGKV3D-11	3D-11*02	QQRSNWH
IGKV3D-11	3D-11*03	QQRSNWH
IGKV3D-15	3D-15*01	QQYNNWP
IGKV3D-15	3D-15*03	QQYNNWP
IGKV3D-20	3D-20*01	QQYGSSP
IGKV3D-20	3D-20*02	QQRSNWH

Fig. S1b

IGKV4		
IMGT gene name	IMGT allele name	aa of CDR-3
IGKV4-1	4-1*01	QQYYSTP

IGKV5		
IMGT gene name	IMGT allele name	aa of CDR-3
IGKV5-2	5-2*01	

IGKV6		
IMGT gene name	IMGT allele name	aa of CDR-3
IGKV6-21	6-21*01	HQSSSLP
IGKV6-21	6-21*02	HQSSSLP
IGKV6D-21	6D-21*01	HQSSSLP
IGKV6D-21	6D-21*02	HQSSSLP
IGKV6D-41	6D-41*01	QQGNKHP

Fig. S1c

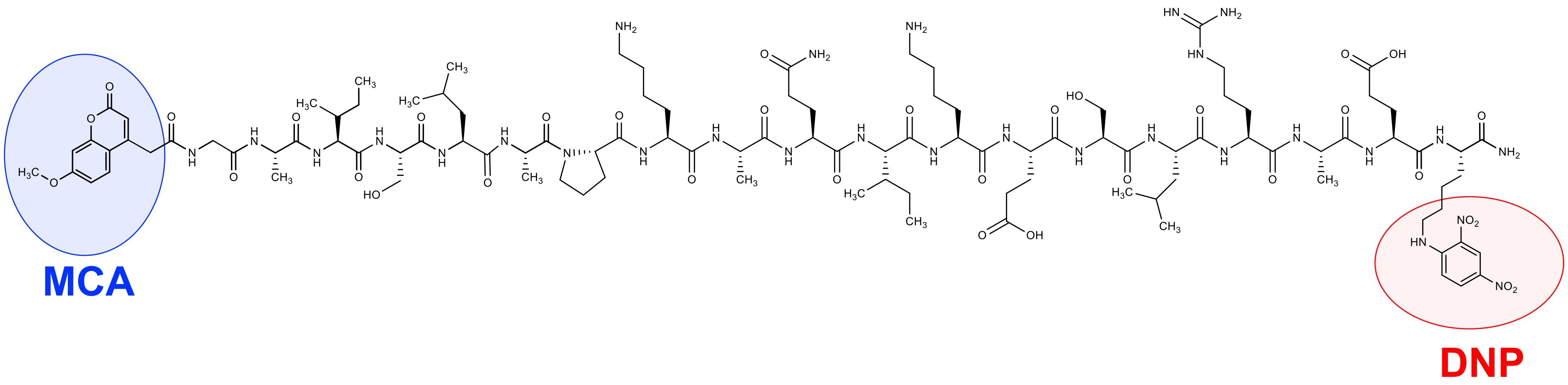

7-MCA–GAISLAPKAQIKESLRAE–K(DNP)–NH_2

(residue 123 = G, residue 140 = E)

Fig. S2

AA sequences of human PD-1 and recombinant PD-1 (rPD1)

(The sequence of 123-140 was selected for FRET-PD1 peptide)

human PD-1 (288mer)

**MQIPQAPWPVVWAVLQLGWRPGWFLDSPDRPWNPPTFSPALLVVTEGDNATFTCSFSNTSESFVLNWYRMSPSNQT
DKLAAFPEDRSQPGQDCRFRVTQLPNGRDFHMSVVRARRNDSGTYLCGAISLAPKAQIKESLRAELRVTERRAEVP
TAHPSPSPRSAGQFQTLVVGVVGGLLGSLVLLVWVLAVICSRAARGTIGARRTGQPLKEDPSAVPVFSVDYGELDF
QWREKTPEPPVPCVPEQTEYATIVFPSGMGTSSPARRGSADGPRSAQPLRPEDGHCSWPL**

Recombinant PD-1 (human): aa 25-167 (with 5'-His-tag, V5 epitope)

**LDSPDRPWNPPTFSPALLVVTEGDNATFTCSFSNTSESFVLNWYRMSPSNQTDKLAAFPEDRSQPGQDCRFRVTQL
PNGRDFHMSVVRARRNDSGTYLCGAISLAPKAQIKESLRAELRVTERRAEVPTAHPSPSPRSAGQFQ**

(The aa sequence of pink color was selected as the portion peptide for FRET peptide)

Fig. S3

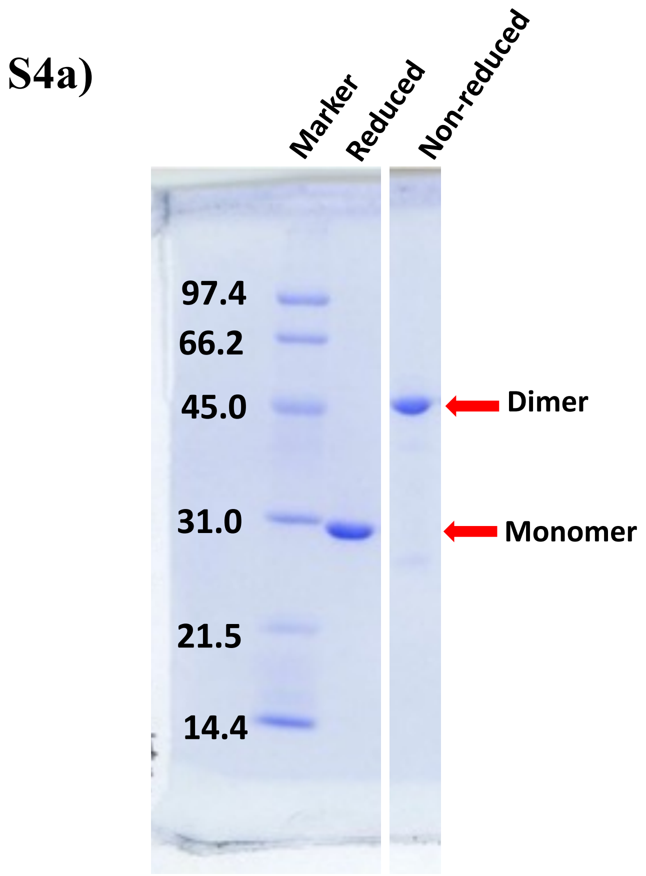

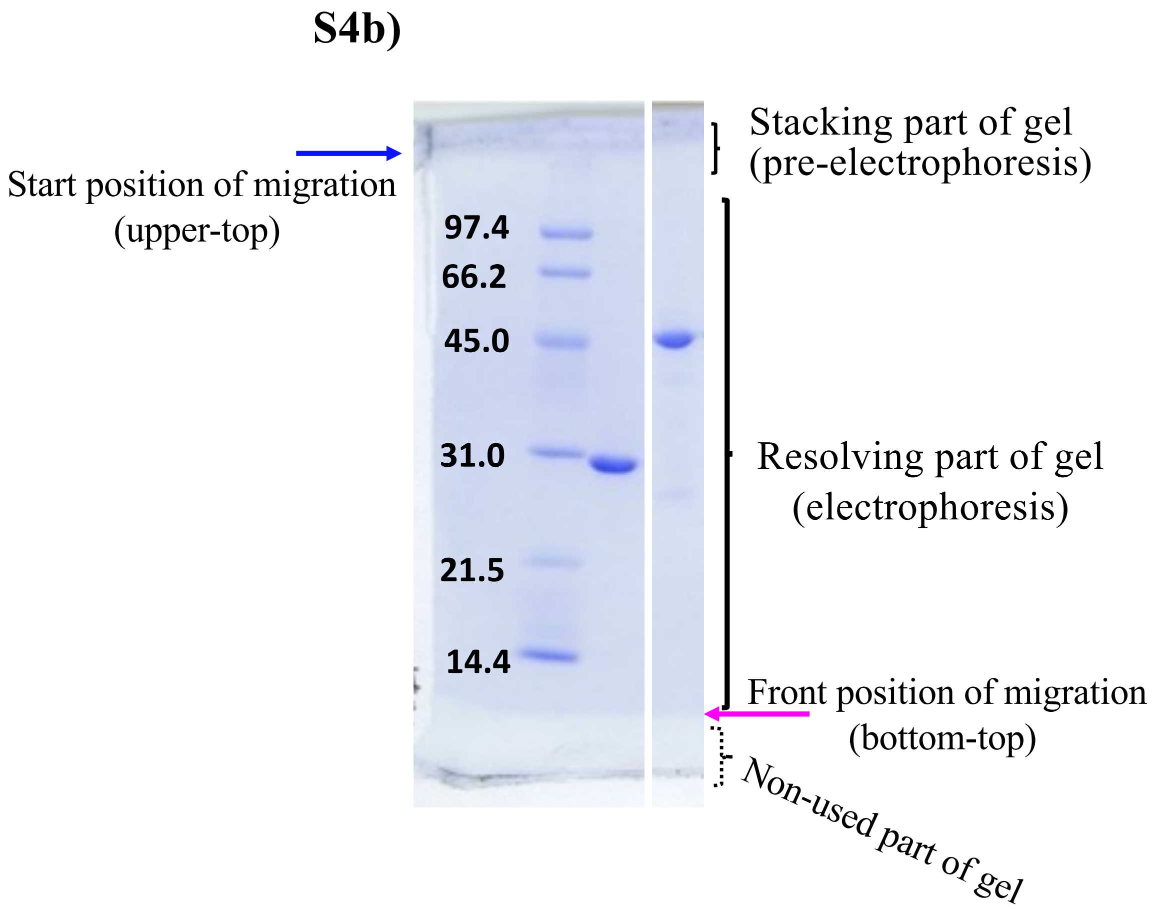

Fig. S4a and S4b
